# Supplementary material for: Data-driven analytics for student reviews in China’s higher vocational education MOOCs: A quality improvement perspective
Source: PLoS One. 2024 Mar 13;19(3):e0298675. doi: 10.1371/journal.pone.0298675 (PMC10936858; doi:10.1371/journal.pone.0298675)
Supplement: Attachment — Submitted filename: Response to reviwers.docx [file pone.0298675.s001.docx]

Dear Editor,

We first thank you and the reviewers for the helpful and constructive comments. We have thoroughly revised our manuscript according to the suggestions of the reviewers. In our revised manuscript, the revised and newly added text have been marked. Below, please find our point-to-point responses to the comments. For the sake of presentation, the journal requirements and reviewers' comments are duplicated in italics.

Best regards,

The authors (Hongbo Li, Huilin Gu, Xue Hao, Xin Yan, Qingkang Zhu)

**Detailed Responses to the academic editor**

1. *When submitting your revision, we need you to address these additional requirements.
   Please ensure that your manuscript meets PLOS ONE's style requirements, including those for file naming. The PLOS ONE style templates can be found at
   https://journals.plos.org/plosone/s/file?id=wjVg/PLOSOne_formatting_sample_main_body.pdf and
   https://journals.plos.org/plosone/s/file?id=ba62/PLOSOne_formatting_sample_title_authors_affiliations.pdf.*

**Response**: We have checked the manuscript’s style and revised the formatting irregularities. We have added figure captions following the paragraph in which the figure is first cited.

2. *We note that the grant information you provided in the ‘Funding Information’ and ‘Financial Disclosure’ sections do not match.
   When you resubmit, please ensure that you provide the correct grant numbers for the awards you received for your study in the ‘Funding Information’ section.*

**Response**: This research was funded by the Soft Science Project of Shanghai Science and Technology Innovation Action Plan (Grant Number 23692113000). When resubmitting, we will check the ‘Funding Information’ and ‘Financial Disclosure’.

3. *Thank you for stating the following financial disclosure:
   [This research was funded by the Soft Science Project of Shanghai Science and Technology Innovation Action Plan (Grant Number 23692113000)].
   Please state what role the funders took in the study. If the funders had no role, please state: ""The funders had no role in study design, data collection and analysis, decision to publish, or preparation of the manuscript.""
   If this statement is not correct you must amend it as needed.
   Please include this amended Role of Funder statement in your cover letter; we will change the online submission form on your behalf.*

**Response**: The funders had no role in study design, data collection and analysis, decision to publish, or preparation of the manuscript. We have amended Role of Funder statement in our cover letter.

4. *We note that you have stated that you will provide repository information for your data at acceptance. Should your manuscript be accepted for publication, we will hold it until you provide the relevant accession numbers or DOIs necessary to access your data. If you wish to make changes to your Data Availability statement, please describe these changes in your cover letter and we will update your Data Availability statement to reflect the information you provide.*

**Response**: We have provided the data. It can be found here: https://github.com/XueHao-23/MOOC-reviews. And we do not make changes to our Data Availability statement.

5. *We note that Figure(s) 1 and 2 in your submission contain copyrighted images. All PLOS content is published under the Creative Commons Attribution License (CC BY 4.0), which means that the manuscript, images, and Supporting Information files will be freely available online, and any third party is permitted to access, download, copy, distribute, and use these materials in any way, even commercially, with proper attribution. For more information, see our copyright guidelines: http://journals.plos.org/plosone/s/licenses-and-copyright.
   We require you to either (1) present written permission from the copyright holder to publish these figures specifically under the CC BY 4.0 license, or (2) remove the figures from your submission:
   a. You may seek permission from the original copyright holder of Figure(s) 1 and 2 to publish the content specifically under the CC BY 4.0 license.
   We recommend that you contact the original copyright holder with the Content Permission Form (http://journals.plos.org/plosone/s/file?id=7c09/content-permission-form.pdf) and the following text:
   “I request permission for the open-access journal PLOS ONE to publish XXX under the Creative Commons Attribution License (CCAL) CC BY 4.0 (http://creativecommons.org/licenses/by/4.0/). Please be aware that this license allows unrestricted use and distribution, even commercially, by third parties. Please reply and provide explicit written permission to publish XXX under a CC BY license and complete the attached form.”
   Please upload the completed Content Permission Form or other proof of granted permissions as an ""Other"" file with your submission.
   In the figure caption of the copyrighted figure, please include the following text: “Reprinted from [ref] under a CC BY license, with permission from [name of publisher], original copyright [original copyright year].”
   b. If you are unable to obtain permission from the original copyright holder to publish these figures under the CC BY 4.0 license or if the copyright holder’s requirements are incompatible with the CC BY 4.0 license, please either i) remove the figure or ii) supply a replacement figure that complies with the CC BY 4.0 license. Please check copyright information on all replacement figures and update the figure caption with source information. If applicable, please specify in the figure caption text when a figure is similar but not identical to the original image and is therefore* *for illustrative purposes only.*

**Response**: We are unable to obtain permission from the original copyright. So we supply replacement figures for Figures 1 and 2 in the revised manuscript. The screenshots of MOOC websites in Figures 1 and 2 (the original images) are taken to show that the raw data consists of both student reviews and student ratings. Instead of Figure 2 and screenshots in Figure 1, we have plotted a figure showing the composition of the raw data. And we have specified in the figure caption text of Revised Manuscript with Track Changes.

6. *Please review your reference list to ensure that it is complete and correct. If you have cited papers that have been retracted, please include the rationale for doing so in the manuscript text, or remove these references and replace them with relevant current references. Any changes to the reference list should be mentioned in the rebuttal letter that accompanies your revised manuscript. If you need to cite a retracted article, indicate the article’s retracted status in the References list and also include a citation and full reference for the retraction notice.*

**Response**: We have reviewed our reference list. There are no retracted papers in the reference list. And we have added two papers according to Reviewer 1’s suggestion.

**Detailed Responses to Reviewer 1**

0. *Thank you for considering PLOS ONE for your submission. In fact, this is a well-written paper. However, there are few comments that need to be addressed to increase the quality of this work.*

**Response**: Thanks very much for your comments. We have revised our manuscript and addressed the issues according to your comments. The detailed descriptions of our modifications are given in the following responses.

1. *The significance of your study is undoubtedly valuable; however, I suggest that the presentation of its importance could be strengthened. Providing a more explicit and focused outline of the study's significance in the introduction would help readers grasp the broader implications of your research from the outset. Consider clearly articulating the real-world problems or gaps in knowledge that your study addresses, and how your findings contribute to filling those gaps. This will not only engage readers more effectively but also emphasize the practical relevance of your work.*

**Response**: In the revised manuscript, we have explained the significance of this study in more detail in the revised fourth paragraph of the Introduction section. Specifically, first, we summarize the gaps in the existing research. The existing studies not only rarely explore students’ differentiated demands implied in the MOOC reviews but also lack attention to MOOCs in higher vocational education. Then, we propose our research content based on these gaps and clarify our contributions.

2. *While your study touches upon practical implications, there is an opportunity to expand on this aspect further. Readers, especially those from non-academic backgrounds, would benefit from a more detailed exploration of how the findings can be applied in real-world scenarios. Consider dedicating a section to explicitly discussing the practical implications of your research. Address questions such as: How can the results inform decision-making in relevant industries or sectors? Are there specific actions that practitioners can take based on your findings? Providing concrete examples or case studies could illustrate the direct impact of your research on practical applications.*

**Response**: In the revised manuscript, we have added a section “6.3 Practical implications” to clarify the practical implications. The practical implications of our study are summarized as follows.

First, this study reveals the specific demands of students for teacher quality, course content, interactivity, etc., which provides higher vocational education institutions and teachers with directions for course improvement. For example, in the course Industrial Robot Practical and Application, whose data have been included in our dataset, the teachers should not only be knowledgeable in theory but also have practical experience operating robots in factories. And they should take into full consideration the students' demands for career skills when preparing the lessons and teach the students more skills to operate the robots correctly.

Second, higher vocational education institutions and MOOC platforms can take action under the guidance of our proposed quality improvement strategies to improve students' satisfaction and increase the attractiveness and retention of the courses. For example, higher vocational schools should establish a teacher team with superb skills and rich theories. Teachers should take into full consideration the differences in learning foundation and ability of students in higher vocational institutions when designing course content. MOOC platforms should support diversified learning resources, including audio, short videos, etc., and enhance the platform's interactivity and personalization features to meet the specific demands of students.

3. *Further related literature can be incorporated to enhance the literature review. This includes but not limited to:
   - Exploring Student Readiness to MOOCs in Jordan: A Structural Equation Modelling Approach. doi: https://doi.org/10.28945/4542
   -The learning behaviours of dropouts in MOOCs: A collective attention network perspective. doi:* [*https://doi.org/10.1016/j.compedu.2021.104189*](https://doi.org/10.1016/j.compedu.2021.104189)

**Response**: In the revised manuscript, we have added and reviewed the above two references in Section 2.1.

**Detailed Responses to Reviewer 2**

1. *In lines 48-50, is there an inclusion relationship between "forum data on MOOC websites" and "MOOC reviews"? The specific meaning of the two is suggested to be distinguished..*

**Response**: In the revised manuscript, we have distinguished the meanings of the two in the introduction. Specifically, forums on MOOC websites support student interaction to learn course content, ask questions, discuss specific topics, and share opinions and experiences, covering a wide range of topics. In this study, MOOC reviews are student evaluations of MOOC resources, which are biased towards students’ experiences of using them. MOOC reviews can be seen as part of forum data on MOOC websites.

2. *It is suggested to add the enlightenments of existing studies to the literature review.*

**Response**: At the end of Section 2, we have summarized the existing studies and analyzed their research gaps to clarify the enlightenment for our research.

3. *In “Experimental results”, almost every part only provides a visual presentation and brief explanation of the data results, and it is suggested to add more specific and in-depth analysis and interpretation, so as to make the strategies proposed later more reliable.*

**Response**: We have complied with this comment. In Section 6.2 of the revised manuscript, we have added more in-depth analysis and interpretation.

4. *What is the relationship between student demand and student satisfaction? How is the conversion between them in this study?*

**Response**: In the revised manscript, we have explained the relationship between student demand and student satisfaction in the second paragraph of Section 1, where we first mention student demand and student satisfaction. The degree of meeting student demands affects student satisfaction with MOOCs. In this study, we use the positive and negative sentiment polarity weights of demands to measure the influence of demand on student satisfaction, and use the KANO model to explain the relationship between the degree of meeting various demands and student satisfaction with MOOCs.
